# Chemical composition, antibacterial efficacy, and antioxidant capacity of essential oil and oleoresin from *Monodora myristica* and *Tetrapleura tetraptera* in Southeast Nigeria

**DOI:** 10.1038/s41598-022-23161-5

**Published:** 2022-11-18

**Authors:** Queency N. Okechukwu, Fabian U. Ugwuona, Chigozie E. Ofoedu, Szymon Juchniewicz, Charles Odilichukwu R. Okpala

**Affiliations:** 1 Department of Food Science and Technology, Michael Okpara University of Agriculture, Umudike, Abia State Nigeria; 2 Institute of Chemical Technology, Ural Federal University Named After the First President of Russia B. N. Yeltsin, Yekaterinburg, Russian Federation; 3 Department of Food Science and Technology, Federal University of Technology, Owerri, Imo State Nigeria; 4 Department of Functional Food Products Development, Wrocław University of Environmental and Life Sciences, 51-630 Wrocław, Poland; 5 UGA Cooperative Extension, College of Agricultural and Environmental Sciences, University of Georgia, Athens, GA 30602 USA

**Keywords:** Biochemistry, Biotechnology

## Abstract

Specific to the West African sub-region, previous studies involving fruit, stem, and bark of *Tetrapleura tetraptera* as well as seeds of *Monodora myristica* have largely focused on phytochemical properties of aqueous and methanolic and ethanolic extracts. To supplement existing information, the chemical composition, antibacterial efficacy (tested against *Escherichia coli* and *Staphylococcus aureus*), and antioxidant capacity (1,1-diphenyl-2-picrylhydrazyl (DPPH∙) radical scavenging, ferric reducing power, and total antioxidant capacity) of essential oil and oleoresin extracted from *T. tetraptera* fruit and *M. myristica* seeds cultivated in Southeast Nigeria, were studied. Essential oil and oleoresin were respectively extracted by steam distillation and aqueous maceration. By way of gas chromatograph mass spectrometry (GC–MS) analysis, the chemical compounds from essential oil and oleoresin from *M. myristica* and *T. Tetraptera* samples totaled 6 and 5, as well as 27 and 16, respectively. Besides the oleoresin of *M. myristica* and the essential oil of *T. tetraptera* showing some resistance against *S. aureus*, the oleoresins seemed highly susceptible to *E. coli*—all of which demonstrated concentration-dependence to the antibacterial inhibition zone. Scavenging DPPH radical, reduction power activity, and total antioxidant capacity increased with essential oil and oleoresin extracts' concentrations, which positions *M. myristica* and *T. tetraptera* spices as very promising for food preservation, especially against autoxidation and microbial spoilage.

## Introduction

Globally, edible plants continue to be of research interest given their natural compounds and medicinal properties that are capable of improving health and preventing/combating diseases^1^. Spices are among such particularly large groups of edible plants with beneficial natural ingredients usually added to foods. Furthermore, the capacity to influence both aroma and taste of foods is largely owed to the presence of essential/volatile oils that comprise terpenes and terpenoids along with various aliphatic hydrocarbons, acids, alcohols, etc.^2^, able to control food spoilage and prolong shelf life^3^. In addition to essential oils, spices show potential health benefits through antimicrobial, antioxidant, antidiabetic, anti-inflammatory, anti-viral, and antiprotozoal capacities. Additionally, the chemical composition and medicinal properties could be influenced by genetics and the type of extraction method^3^. In addition to their abundance from rural to urban areas, spices from African flora have helped in the discovery of novel drugs useful in biomedicine, which have helped to promote health and tackle diseases such as cancer, tumors, etc. Examples of indigenous spices in Africa include black pepper (*Xylopia aethiopica*)*,* West African pepper (*Piper guineese), Mentha piperita, Ocimum gratissimum, Tetrapleura tetraptera* and *Monodora myristica.* Unlike the exotic types, most of the above-named spices are wholly used and continue to receive research attention given the reported phytochemical constituents, antioxidant, and antimicrobial capacities^1,3–7^.

On one hand, the *T. tetraptera* is a deciduous perennial flowering medicinal plant of West African origin belonging to the pea family(Fabaceae), and commonly known as *aridan* and *yanayan* amongst the Yoruba and Urhobos respectively in Nigeria and *prekese* in Ghana^4,5^. The essential oil present in *T. tetraptera* provide a distinctive pleasant fragrance and aroma, making its dry fruit a popular seasoning spice in South-eastern Nigeria^5^. Ghanaians believe that *T. tetraptera* fruits contain multivitamins, useful in the management of jaundice, inflammation, convulsion, fever, and leprosy^1,4,8^. The phytochemical constituents of *T. tetraptera* fruits provide medicinal attributes demonstrated by molluscicidal, antimicrobial, antioxidant, anticonvulsant, anti-inflammatory, antimalarial, antidiabetic, and anticancer properties^8–11^. Microbiological studies show the extract of *T. tetraptera* exhibits excellent antimicrobial activity against Gram-positive and Gram-negative bacteria^4,12^. On the other hand, the *M. myristica*, also known as African/calabash nutmeg, is a perennial plant of the family Annonaceae, which thrives in the tropics of West Africa and the Caribbean. Common names of this plant in Nigeria include ehuru, ariwo, ehiri, airama, and awerewa, lubushi^6^. The seed is rich in oil and is of great value due to its medicinal and nutritional qualities^3^. The fruits and seeds are used as stimulants, stomachic, against headaches, and sores, and as a natural insect repellent. The essential oil contains compounds like α-phellandrene, α-pinene, myrcene, limonene, and pinene. The pleasant aroma, like nutmeg, makes this spice useful in the preparation of traditional dishes in Nigerian communities^6^. Spice seed extracts are believed to possess both antioxidant^1,7^ and antimicrobial properties^3^. *M. myristica* could be effective in the treatment of stomach aches, febrile pains, eye diseases, and hemorrhoids^13^, while *Tetrapleura tetraptera* helps to tackle diabetes mellitus, arthritis, hypertension, epilepsy, and asthma^5^.

Both *T. tetraptera* and *M. myristica* are among such common indigenous spices in Nigeria that are under-utilized/-valued, despite their sharp aroma and flavor that is highly perceivable by the sense of smell^3,4^. However, specific to West Africa sub-region, previous studies involving fruit, stem, and bark of *T. tetraptera* as well as seeds of *M. myristica* have largely focused on phytochemical properties of aqueous and methanolic and ethanolic extracts. There is a need, therefore, for further investigations into the essential oil and oleoresin of this *T. tetraptera* and *M. myristica,* specifically their chemical composition, antioxidant, and antibacterial properties and such studies would require respective activities of steam distillation maceration technique, which would help unravel their potential/relevance in the various communities in southeast Nigeria where they serve as a natural preservative to prevent food spoilage. To supplement existing information, this current work investigated the chemical composition, antibacterial efficacy, and antioxidant capacity of essential oil and oleoresin extracted from *T. tetraptera* fruit and *M. myristica* seeds cultivated in Southeast Nigeria. Specifically, the essential oil and oleoresin respectively extracted by steam distillation and aqueous maceration were subsequently subject to analytical tests in adherence to the relevant institutional guidelines. Furthering the knowledge and understanding underpinning the capacities of these extracted essential oil and oleoresin to tackle food spoilage challenges is warranted to help consolidate the product development potential of both *T. tetraptera* fruit and *M. myristica* seeds.

## Materials and methods

### Schematic overview of the experimental program

**Figure 1:**
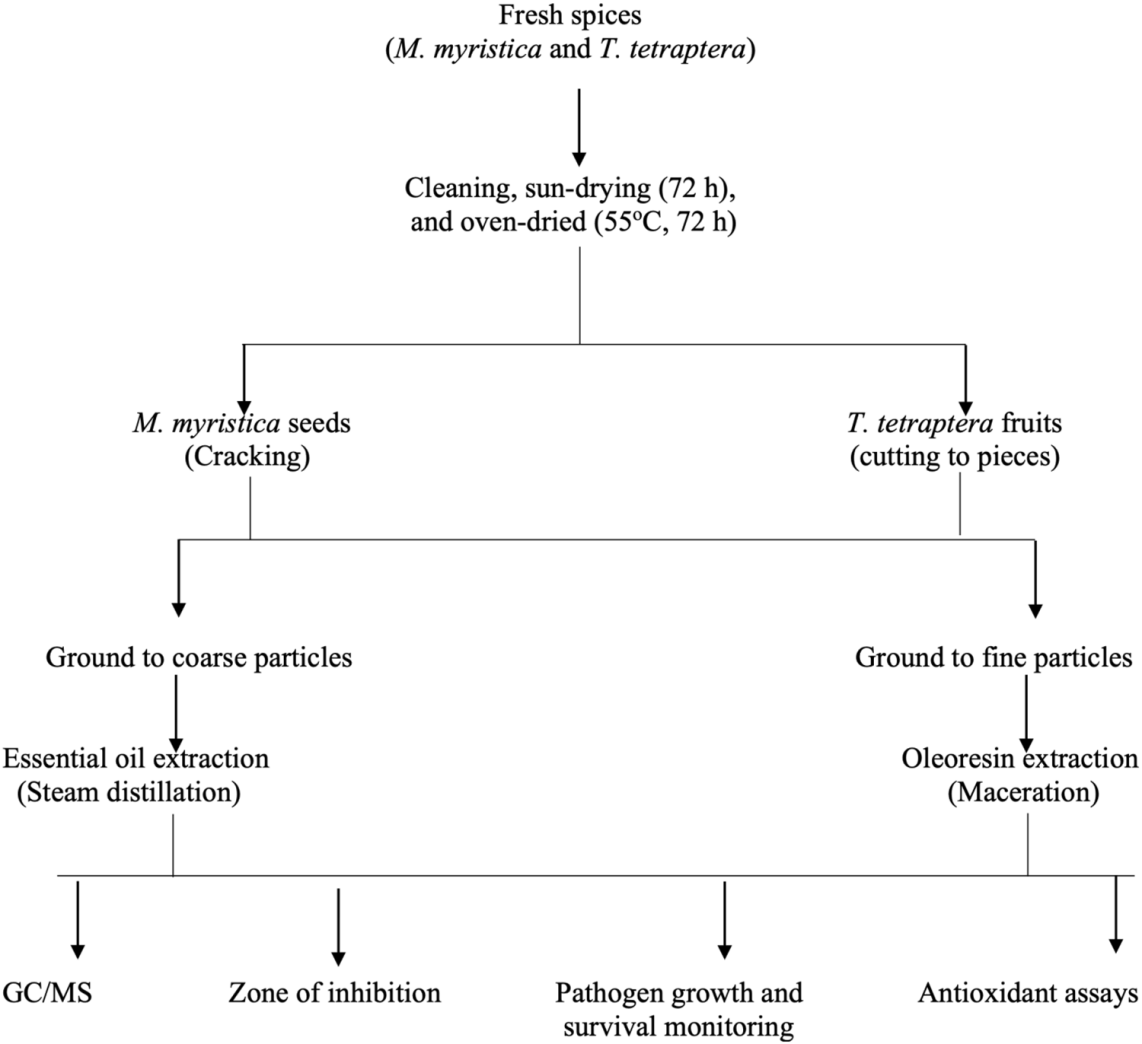
The schematic overview of the experimental program of this current study, which depicts the major stages from procurement of plant materials, processing of plant parts extraction processes, prior to the subsequent analytical measurements.

Figure 1 shows the schematic overview of the experimental program of this current study, which depicts the major stages from procurement of plant materials, and processing of plant parts extraction processes, before the subsequent analytical measurements. For emphasis, this conducted research was directed to understand how the extracted essential oil and oleoresin from *T. tetraptera* fruit and *M. myristica* seeds would thrive specific to the context of chemical composition, antibacterial efficacy (tested against *Escherichia coli* and *Staphylococcus aureus*), and antioxidant capacity (1,1-diphenyl-2-picrylhydrazyl (DPPH∙) radical scavenging, ferric reducing power (FRAP), and total antioxidant capacity (TAC). Additionally, the analytical measurements were carried out independently using the different essential oil and oleoresin samples obtained from individual batches of *T. tetraptera* and *M. myristica* seeds. Importantly, the analytical measurements conducted were in adherence to the relevant guidelines prescribed by the Department of Food Science and Technology, Michael Okpara University of Agriculture, Umudike, Abia State, Nigeria. Furthermore, all chemicals and reagents used in this work, which were procured from reputable registered chemical retailers, were of analytical grade standard.

### Procurement and processing of plant materials

**Figure 2:**
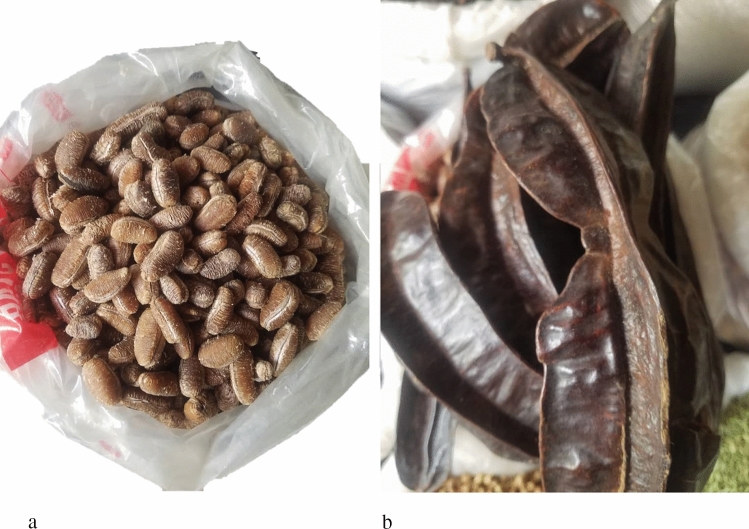
Photo images of *Monodra myristica* (African nutmeg) seeds (**a**) and *Tetrapleura tetraptera* fruits (**b**).

Fresh *T. tetraptera* fruit (approx. 1000 g) and *M. myristica* seeds (approx. 800 g) were procured as two separate batches from a local market in Owerri Imo State, in the Southeast of Nigeria. Furthermore, Fig. 2 shows the photo images of *M. myristica* seeds (Fig. 2a) and *T. tetraptera* fruits (Fig. 2b). To further confirm the samples, the samples were identified at the Department of Plant Biotechnology of Michael Okpara University of Agriculture, Umudike, Abia State, Nigeria. The processing of fresh *T. tetraptera* fruit and *M. myristica* seeds was carried out following the method described by^14^ with modifications. Specifically, the spices were washed with clean running water, sun-dried (72 h), and then oven dried using a Memmert UN30 (Memmert GmbH + Co. KG, Schwabach, Germany) oven at 55 °C for 72 h. The *M. myristica* seeds were cracked manually to recover the nibs, whereas the fruits of *T. tetraptera* were cut into small pieces, and both were kept in ambient conditions until required for further processing.

### Extraction processes of essential oil and oleoresin

Already cracked *M. myristica* seeds, and small pieced cut *T. tetraptera* fruit samples were individually ground into coarse and fine particles using an attrition type mill K-Tron type T-35volumetric feeder (K-Tron Corp. Pitman NJ USA). The coarse particles were kept for essential oil extraction, whereas those of fine powder was for oleoresin extraction. On one hand, essential oil extraction involved the steam distillation method as described by Chemat et al.^15^ with slight modification. Briefly, the conventional type steam distillation apparatus with a cylindrical Pyrex body supported by Teflon grid at its lower end, with a rheostat-controlled heating facility, was used. The essential oil was dried over anhydrous sodium sulfate (Na_2_SO_4_) and stored in airtight amber bottles at 4 °C until used. On the other hand, the oleoresin extraction involved the maceration technique modified from Fernández‐Ronco et al.^16^. For each spice, 200 g of finely ground spice was macerated in 400 ml of distilled water in a 1000 ml conical flask and tightly corked. This was shaken vigorously at 30-min intervals for 3 h, after which the supernatant was decanted and the residue was filtered out. After extraction, the supernatant from each spice was collected and evaporated at 60 °C in a stifling air oven (Memmert UN30, Memmert GmbH + Co. KG, Schwabach, Germany) for 8 h. The oleoresins were stored in airtight amber bottles and kept in the refrigerator until used.

### Analytical measurements

#### Determination of chemical composition

The chemical composition of the crude essential oil and oleoresin samples was determined following the routine method employed in the laboratory of the Department of Food Science and Technology, Michael Okpara University of Agriculture, Umudike, Abia State, Nigeria. This involved the gas chromatograph mass spectrometry (GC–MS) analysis, specifically the Agilent 6890 gas chromatograph (Supelco, Bellefonte, PA, USA) equipped with an on-column automatic injector, flame ionization detector, and HP 88 capillary column (100 m × 0.25 μm film thickness). The carrier gas was helium at a constant flow rate of 1.0 mL/min. The detector was maintained at 250 °C and both injectors at 220 °C with integrator chart speed at 2 cm/min. The initial column temperature was set at 100 °C (hold time =  ~ 2 min) to the final temperature of 180 °C at a rate of 50 °C/min, the volume injected was 1.0 μL and the split ratio was 50:1. Total chromatogram was auto-integrated by Shem-Station, and emergent chemical constituents were identified by comparison with both published mass spectral database (NIST02.L) and available literature data.

#### Determination of antibacterial efficacy

##### Measurement of inhibition zone

The agar well diffusion method according to Balouiri, Sadiki, and Ibnsouda^17^ was used. The microorganisms (0.1 mL) from previously prepared diluents were spread on previously prepared nutrient agar in standard Petri dishes in triplicates. Wells of 2 mL diameter and 0.5 mL depth were created using a sterile bore. The essential oil and oleoresin samples were introduced using a sterile micropipette, after which Petri dishes were stored at 37 °C for 24 h in an incubator to observe and measure the zone of inhibition (mm) of microbial growth caused by the aforementioned substances.

##### Monitoring of pathogen growth and survival

Staphylococcus *aureus* ATCC 27840 and *Escherichia coli* 0157, routinely used for the antibacterial assay, were obtained from the microbiology department of the University of Nigeria Nsukka, Nigeria. Substrates were refrigerated at 4 °C for 5 days and the growth and survival of the pathogens were monitored using the Plate colony-counting method. Using 1.0 mL from each pathogen *Escherichia coli*, and *Staphylococcus aureus,* suspended to a density of 10^5^, were inoculated into a flask with nutrient agar. Extracts diluted in 0.1% buffered peptone water to appropriate concentrations, subsequently mixed thoroughly, and aseptically poured onto a petri dish. Colonies were manually counted periodically within 5 days of incubated storage at 37 °C.

#### Determination of antioxidant capacities

##### Free radical scavenging activity

Free radical scavenging activity of the already extracted essential oil and oleoresin were measured using the radical 1, 1-diphenyl-2-Picrylhydrazyl (DPPH) as described by Shah et al.^18^ with slight modifications. This involved dissolving ~ 25 mg of DPPH in 100 mL methanol, and thereafter storing at 20 °C till needed. From the working solution obtained from the DPPH stock solution by methanol, 1 mL was added to 100 μL of various concentrations of test samples, incubated for 45 min, thoroughly shaken, and then left in the dark under ambient conditions. Ascorbic acid served as the standard, scavenging effect was calculated by Eq. (1):

1 $$\%\;Scavenging\;Effect=\frac{({A}_{DPPH}-{A}_{EOIL})}{{A}_{DPPH}}*100$$

A_DPPH_ = absorbance of DPPH at 517 nm and A_EOIL_ = absorbance of oils/resins at 517 nm.

##### Iron (III) to iron (II)-reducing power assay (FRAP)

The ability of essential oil and oleoresin to reduce Fe^3+^ to Fe^2+^ was assessed by the method of Hinneburg, Dorman, and Hiltunen^19^ with slight modifications. A 2-mL aliquot of sample was added to 2.5 mL of sodium phosphate buffer (0.2 M, pH 6.6, 2.5 mL) containing potassium ferricyanide [K_3_Fe (CN)_6_] (1%, 2.5 mL). After the reaction mixture had incubated at 50 °C for 25 min, then 2.5 mL of trichloroacetic acid (10%) was added and centrifuged at 3000 rpm for 10 min. Subsequently, the supernatant (5 mL) was mixed with 2.5 mL of water, and 0.5 mL of 0.1% aqueous FeCl_3_, followed by an absorbance reading at 700 nm. Gallic acid was used as a standard reference compound.

#### Phosphomolybdate assay (total antioxidant capacity)

The total antioxidant capacity (TAC) of the essential oil and oleoresin was determined by the phosphomolybdate method of Jan et al.^20^ with slight modifications. An aliquot (~ 0.1-mL) of the sample was mixed with 1 mL reagent solution (0.6 M sulfuric acid, 28 mM sodium phosphate, and 4 mM ammonium molybdate), thereafter covered and incubated in a water bath at 85 °C for 75 min. When samples had been cooled, the absorbance was measured at 765 nm. The ascorbic acid was used as a standard reference compound. The TAC was estimated using the Eq. (2) below:

2 $$TAC\; \left(\%\right)=\frac{(Abs.\;of\;control-Abs.\;of\;sample)}{Abs.\;of\;control}*100$$

### Statistical analysis

The data of three replicates from different samples were subjected to factorial approach analysis of variance (ANOVA). Results were represented as means ± standard error, with mean separation conducted using Fisher’s least significant difference (LSD) per measured variable. The probability level was statistically significant at p < 0.05 (95% confidence). Minitab 2018 software (Minitab, LLC, USA) was used to run the data.

## Results and discussion

### Chemical composition of essential oil and oleoresin

**Figure 3:**
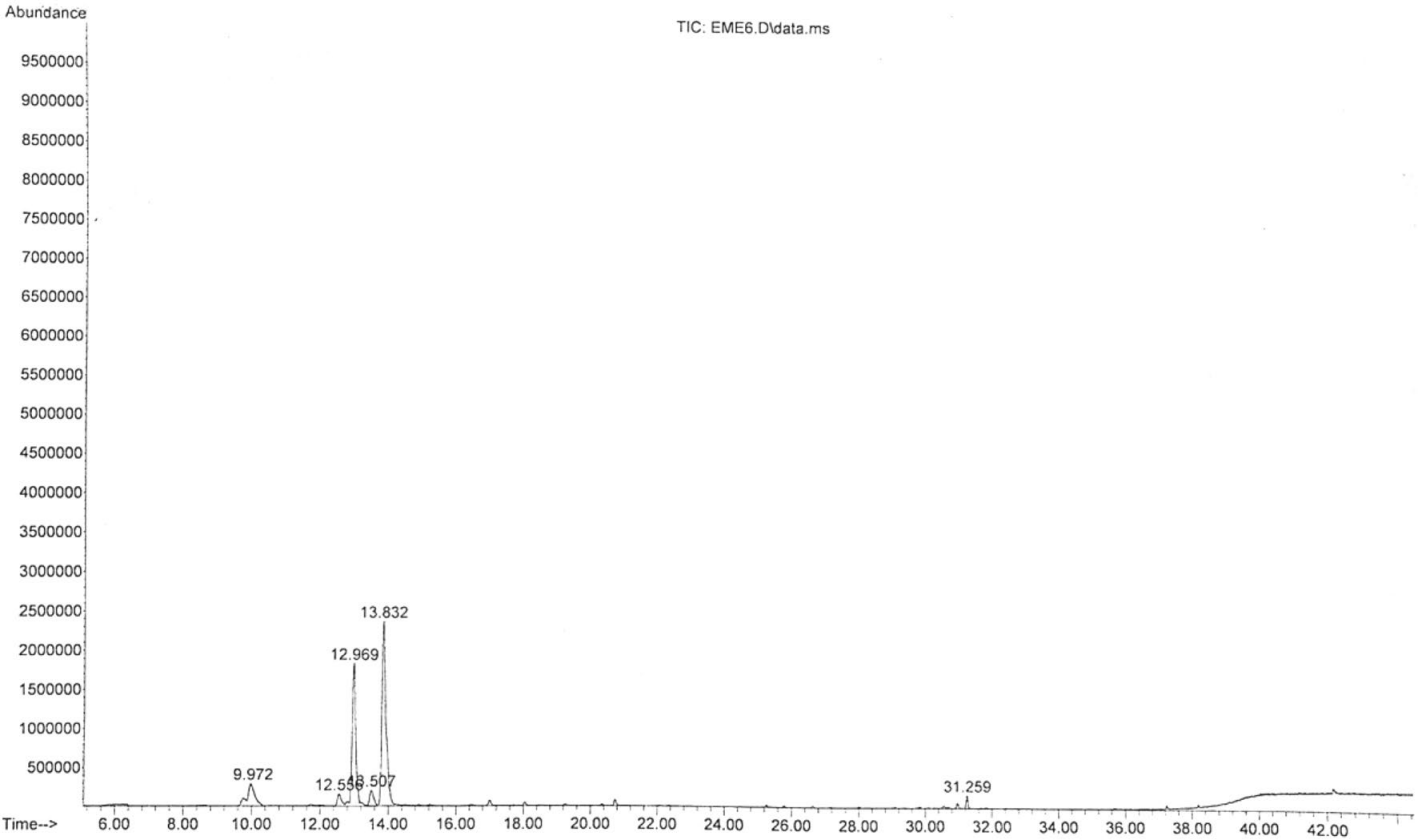
GC–MS chromatogram of *Monodora myristica* essential oil.

**Figure 4:**
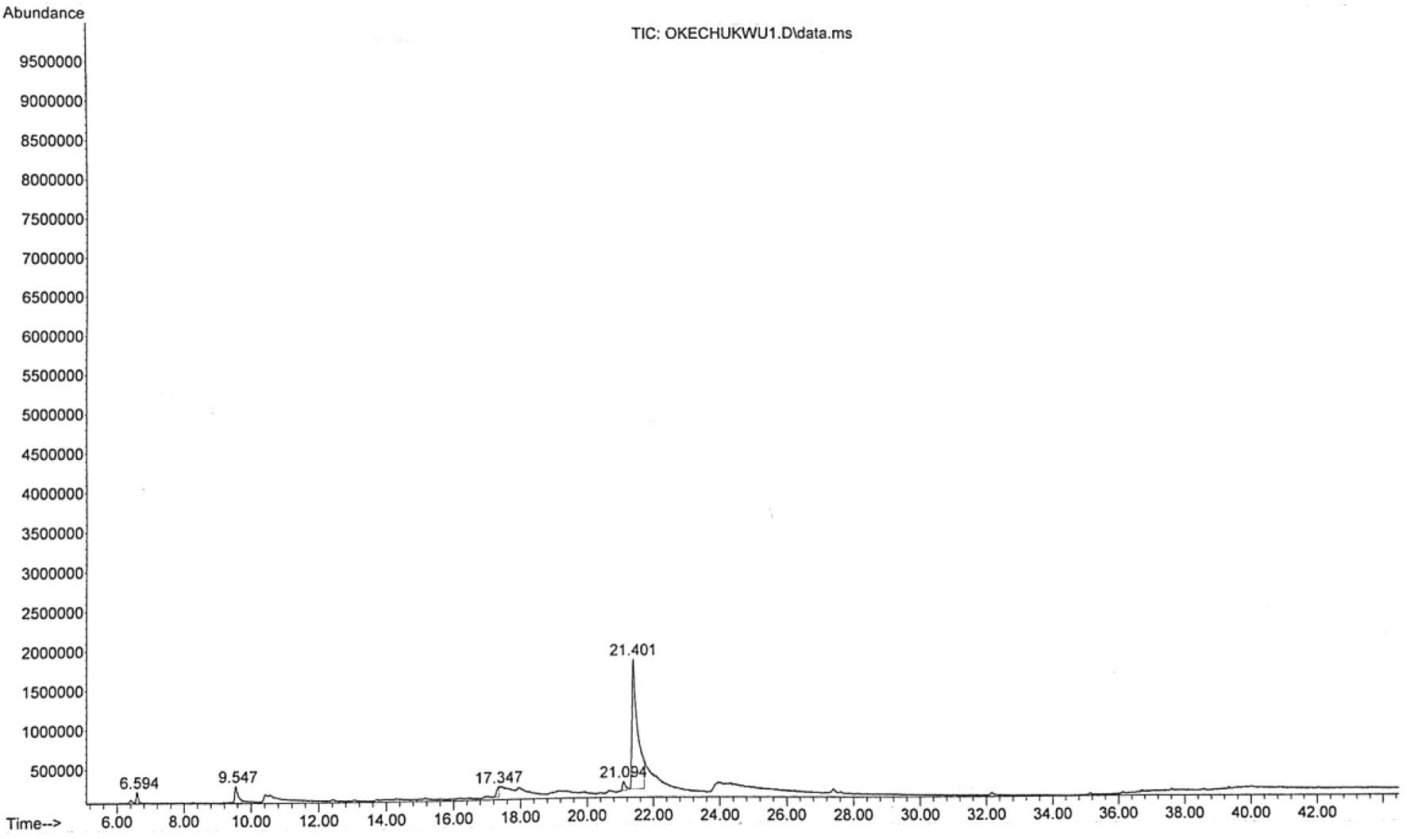
GC–MS chromatogram of *Monodora myristica* oleoresin.

**Figure 5:**
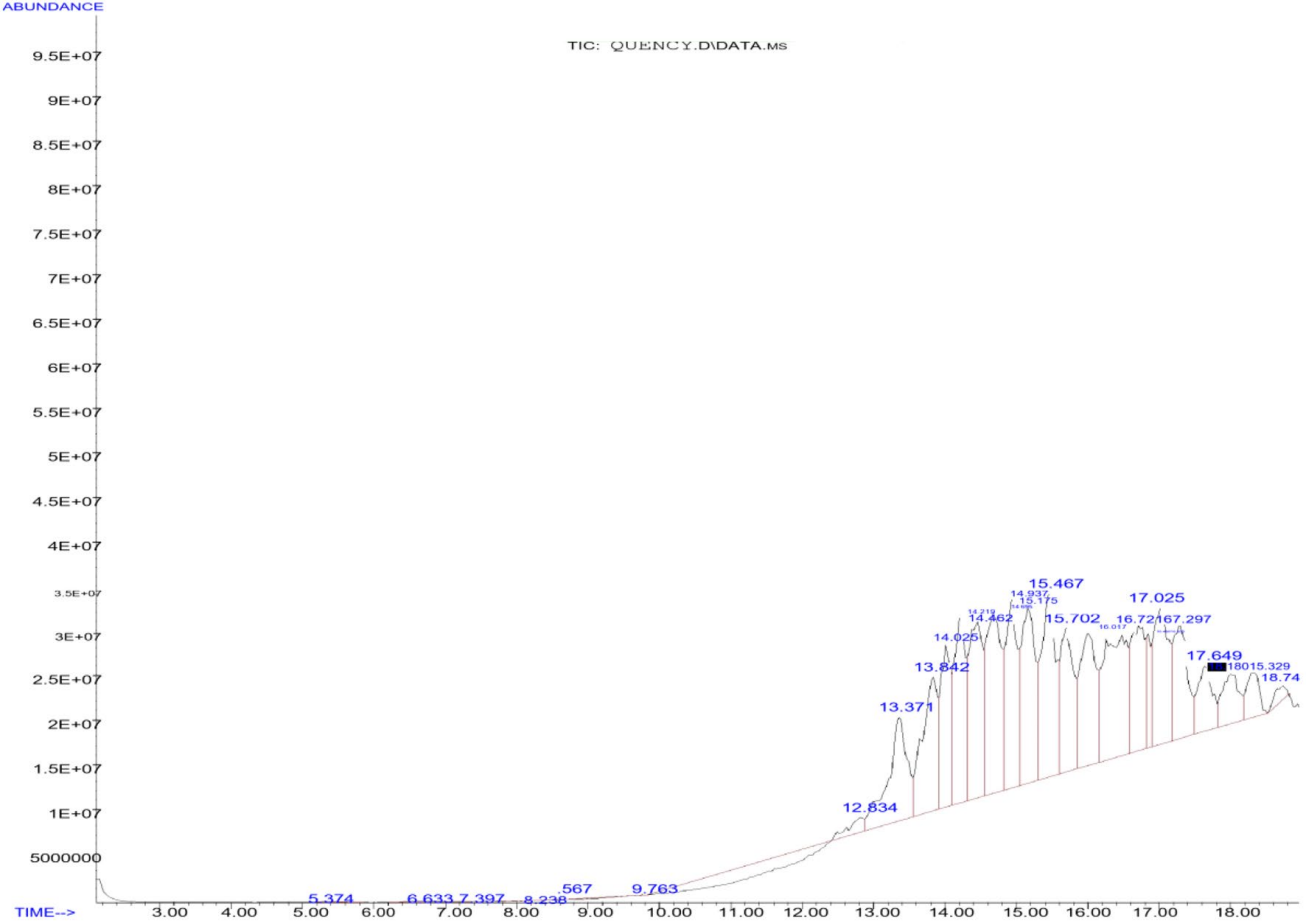
GC–MS chromatogram of *Tetrapleura tetraptera* essential oil.

**Figure 6:**
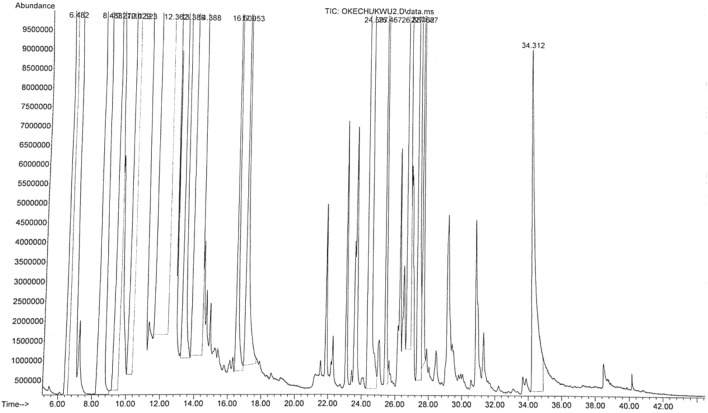
GC–MS chromatogram of *Tetrapleura tetraptera* oleoresin.

The main chemical compounds of essential oil and oleoresin of *M. myristica* and *T. tetraptera* are shown in Tables 1 and 2, with their respective GC–MS chromatograms shown in Figs. 3, 4, 5 and 6. Specific to *M. myristica,* the identified chemical compounds predominantly monoterpenes respectively totalled 6 and 5 in essential oil and oleoresin. Chemical compounds in essential oil would include p-cymene at 50.58% as peak, followed by α-phellandrene at 32.09%, with α-pinene at 8.71% as least, whereas in oleoresin would include, 2-acetyl cyclopentanone (85.42%) as predominant, with α-phellandrene (6.40%) as least. Only δ-cadinene (1.56%) appeared in the sesquiterpene but very little. Previous reports about different extracts of *M. myristica* specific to Nigeria obtained variations in chemical constituents. For example, a report of seed oil of *M. myristica* from southwest Nigeria obtained major constituents like α-phellandrene epoxide (3.02%), carvacrol (2.09%), and δ-cadinene (2,21%)^21^, as well as p-cymene (31.5%), α-phellandrene (18.1%), α-pinene (6.1%), β-pinene (5.1%)^22^. Elsewhere also, essential oil of *M. myristica* obtained by steam distillation showed chemical constituents like α-phellandrene (50.4%), α-pinene (5.5%), myrcene (4.35%), and germacrene-d-4-ol (9.0%)^23^, whereas another seed oil reported elsewhere showed chemical constituents like germacrene-d-4-ol (25.48%), tricyclo [5.2.1(1,5)] dec-2-ene (13.35%), δ-cadinene (11.09%) and linalool (15.10%), and from their stem barks with chemical constituents like γ-cadinene (31.31%), α-elemene (17.98)^13^. In a neighbouring country of Chad and Cameroon, the essential oils extracted by hydrodistillation from fruits of *Xylopia aethiopica* (Dunal) A. Rich, *Xylopia parviflora* (A. Rich) Benth.) and *M. myristica* (Gaertn) showed α-phellandrene (Chad = 52.7%; Cameroon = 67.1%) as a major constituent^24^. Specific to *T. Tetraptera*, the chemical compounds of essential oil and oleoresin respectively totalled 27 and 16 (Tables 2 and 3, as well as Figs. 2, 3, 4 and 5). On one hand, the essential oil comprised carboxylic acids, phenolic acids, and esters, for example, 1-naphthalenecarbonitrile, 2-methoxy- (8.26%), dodecanoic acid, 1-(hydroxymethyl)-1,2-ethanediyl ester (7.90%), benzenamine, 4-methoxy-*N*-(triphenylphosphoranylidene)- (7.78%), 4-dibenzofuranamine (7.08), etc. On the other hand, the oleoresin comprised monoterpenes, for example, γ-terpinene (25.63%), linalool (20.74%), caryophyllene (9.68%), β-pinene (9.38%), 2-carene (6.98), γ-elemene (5.47%), etc. Terpenes were not found in the essential oil of *T. tetraptera*. The essential oil extracted from *T. tetraptera* fruits via hydro-distillation by Erukainure et al.^4^ largely showed acetic and carboxylic acid, with little traces of terpenes. Probably, the presence of linalool might account for the plant’s peppery nature, whereas both γ-terpinene and α-thujene might account for the flavor/fragrance. Elsewhere, the root and stem ethanol extract of *T. tetraptera* spice respectively obtained 70.14%^25^ and 32.01%^10^. Polyphenols like eugenol, quercetin, rutin, and tyrosol, according to Moukette et al*.*^1^, were considered 5.88–10.79-fold more concentrated in the water and ethanolic extracts of the fruits compared to the barks. Furthermore, α-phellandrene and α-pinene has been identified as a major chemotype for *M. myristica* seeds, whereas citral and linalool as major chemotypes of *T. tetraptera*^1,4,21,22^. Such factors as environmental influences, experimental conditions, place of origin, post-harvest handling of the fruits, adaptive metabolism of plants, and plant part analyzed might be responsible for the variations in the chemical compounds that have been detected in *M. myristica* and *T. tetraptera* samples at this current work.

**Table 1:** Major chemical compounds of essential oil and oleoresin found in *Monodora myristica.*

S/N	Chemical compounds	*Monodora* essential oil (%)	*Monodora* oleoresin (%)	Retention time (min)
1	l-α-Pinene	–	2.60	6.594
2	α-Phellandrene	–	6.40	9.547
3	α-Pinene	8.71	–	9.972
4	β-Myrcene	3.17	–	12.556
5	α-Phellandrene	32.09	–	12.969
6	2-Carene	3.90	–	13.507
7	p-Cymene	50.58	–	13.832
8	1,2-(Methylenecyclopropyl) Cyclopentene	–	2.65	17.347
9	2,4-Deimethyl-1,3-Cyclopentanedione	–	2.93	21.094
10	2-Acetylcyclopentanone	–	85.42	21.401
11	δ-Cadinene	1.56	–	31.259

**Table 2:** Major chemical compounds of essential oil and oleoresin found in *Tetraplura tetraptera.*

S/N	Chemical compounds	Retention time	Area (%)	
			Essential oil	Oleoresin
1	1-Oxa-4azaspiro [4.5] decan-4-oxyl,3,3-dimethyl-8-oxo-	5.384	0.05	
2	α-Thujene	6.482		3.87
3	1-Octyn-3-ol	6.623	0.13	
4	1,3-Cyclohexadiene, 5-ethyl-	7.384	0.01	
5	1,3-Cyclopentadiene, 5,5-dimethyl-	8.229	0.01	
6	β-Pinene	8.483		9.38
7	Cyclopentyl acetylene	8.567	0.01	
8	β-Phellandrene	9.272		2.47
9	1H-Imidazole-2,4(3H,5H)-dione, 5-phenyl-5-(2-pyridinyl)-	9.750	0.05	
10	(+)-4-Carene	10.129		2.81
11	Terpinolene	10.523		2.68
12	γ-Terpinene	12.362		25.63
13	Tetrasiloxane, decamethyl-	12.820	3.69	
14	3-Bromobenzoic acid, 10-undecenylester	13.384	5.85	
15	Cyclotrisiloxane, hexamethyl-	13.835	5.90	
16	2-Carene	13.388		6.98
17	Benz(cd)indol-2(1H)-one, 1-methyl-	14.032	4.68	
18	Carazolol	14.229	6.03	
19	Linalool	14.388		20.74
20	4-Dibenzofuranamine	14.454	7.08	
21	Dodecanoic acid, 1-(hydroxymethyl) -1,2-ethanediyl ester	14.708	7.90	
22	Dodecanoic acid, 1-(hydroxymethyl)-1,2-ethanediyl ester	14.933	6.29	
23	Dodecanoic acid, 1-(hydroxymethyl)-1,2-ethanediyl ester	15.187	6.97	
24	Benzenamine,4-methoxy-N-(triphenylphosphoranylidene)-	15.468	7.78	
25	Phenoxazine	15.694	5.40	
26	Phenoxazine	16.004	6.23	
27	1-Naphthalenecarbonitrile, 2-methoxy-	16.483	8.26	
28	Terpinen-4-ol	16.509		1.42
29	1,2-Cyclohexanedicarboxylic acid, 4-bromophenyl ethyl ester	16.736	5.03	
30	Benzenamine,4-methoxy-N-(triphenylphosphoranylidene)-	16.849	1.54	
31	2,5-Cyclohexadien-1-one, 4-(phenylimino)-	17.018	5.98	
32	α-Terpineol	17.053		1.58
33	Fumaric acid, 2,3-dichlorophenyl isohexyl ester	17.299	4.84	
34	2-Hydroxycarbazole	17.638	2.74	
35	4-Nitrophenyl laurate	18.004	2.42	
36	Dodecanoic acid, 2,4,5-trichlorophenyl ester	18.342	1.71	
37	Dodecanoic acid, 1,2,3-propanetriy l ester	18.736	0.91	
38	Caryophyllene	24.597		9.68
39	Humulene	25.467		1.30
40	γ-elemene	26.887		5.47
41	α-Farnesene	27.462		3.59
42	δ-Cadinene	27.687		0.92
43	β-Sinensal	34.312		1.48

**Table 3:** Antibacterial zone of inhibition (mm) of *Mondora myristica* and *Tetrapleura tetraptera* essential oils and oleoresins.

Spices	Concentration (µg/mL)	*Escherichia coli*	*Staphylococcus aureus*
MM oleresin	50	12	–
	75	13	–
TT oleoresin	50	13	–
	75	15	8
MM essential oil	50	13	11
	75	16	13
TT essential oil	50	–	–
	75	11	–

M.M = *Monodora myristica,* T.T = *Tetrapleura tetraptera.*

### Antibacterial efficacy of essential oil and oleoresin

As mentioned earlier in this paper, there is evidence that the extract of *T. tetraptera* would exhibit very promising antimicrobial activity against Gram-positive/-negative bacteria^4,12^. Table 3 shows the antibacterial inhibition zone of essential oils and oleoresins of *M. myristica* and *T. tetraptera*, which appeared concentration-dependent to the bacterial pathogens. Whereas oleoresin obtained the highest susceptibility to *E. coli*, those of *M. myristica* together with the essential oil of *T. tetraptera* showed some resistance to *S aureus*. Considering that the inhibition zone qualitatively deciphers the potentials of an antibacterial agent, the essential oil of *M. myristica* at concentration of 75 µg/mL obtained the highest zone (16 mm) against *E. coli* whereas the essential oil from *T. tetraptera* obtained the least activity. Elsewhere, inhibition zones of 8 mm have been reported and 13 mm for 2.5 mg/mL and 5.0 mg/mL, respectively, of essential oil *M. myristica* against *E. coli*^3^*,* whereas 11 mm for 2 mL also of *M. myristica* essential oil against *E. coli*^26^. Another study showed *T. tetraptera* stem extract against two *Salmonella* strains, with a remarkably high inhibition zone (> 20 mm diameter)^10^, besides the absence of carvone component believed as responsible for the weak activity of oleoresins^27,28^. Comparatively, the promising antibacterial activity of essential oil from *M. myristica* might be dependent on factors like chemical composition and its solubility^3^. The extraction process, cell wall composition of microbial entity, environmental conditions, chemotype of the plant parts used (which often influences biosynthetic pathways), as well as other physiological properties might also potential factors that contribute to the differences in the antibacterial activity.

Natural compounds (terpenes inclusive), which are secondary metabolites from plants, are responsible for the defense against pathogens^29^ and promising in controlling pathogenic/spoilage microbial entities in foods. The survival of the *E*. *coli* and *S. aureus* population involving different concentrations of *Mondora myristica* and *Tetrapleura tetraptera* essential oil and oleoresin can be seen in Table 4. Of both spices, the survival of *E*. *coli* and *S. aureus* population appeared significantly higher (p < 0.05) in the essential oil above those of oleoresin. Within three (3) days of storage, specific to concentrations 25 and 50 µg/mL of essential oil from *T. tetraptera,* the pathogen population increased markedly (p < 0.05) at 2.15 × 10^5^ and 1.53 × 10^5^ CFU/mL (*E. coli*) and 2.5 and 2.03 × 10^5^ CFU/mL (*S. aureus*), respectively. Within 3 days of storage, however, no bacteria proliferated in *T. tetraptera* oleoresin as well as *M. myristica* essential oil and oleoresin. By day 5, the *T. tetraptera* essential oil respectively showed 7.05 and 6.10 × 10^5^ CFU/mL for *E. coli* and *S. aureus* (Table 5). However, *S. aureus* obtained 6.51 and 4 × 10^7^ CFU/mL at 25 and 50 µg/mL for *M. myristica* essential oil, significantly different(p < 0.05) from 1.5 × 10^4^ CFU/mL at 25 µg/mL for *T. tetraptera* oleoresin. Gram-positive bacteria are believed to reveal more susceptibility to antibacterial compounds compared to Gram-negative bacteria^28,30^, given the lipoproteins and lipopolysaccharides present in the cellular wall/barrier-like structure that restricts the entry of hydrophobic compounds^31^. In essential oil, however, presence of terpenes should provide the major antibacterial capacity. More so, some simple phenols might appear in the form of phenylpropanoids^32^. Besides essential oils and oleoresins of this current work being concentration-dependent, their complex mix of several (minor) chemical components would assert multifunctional synergistic effects^33,34^.

**Table 4:** Survival of *Escherichia coli* and *Staphylococcus aureus* population (10^7^ × CFU/mL) involving different *Mondora myristica* and *Tetrapleura tetraptera* essential oils and oleoresin concentrations.

Spices	Storage period (day)	*Escherichia coli*		*Staphylococcus aureus*	
		Concentration (µg/mL)			
		25	50	25	50
M.M; essential oil	1	2 × 10^7K^ ± 0	4 × 10^7I^ ± 0	7 × 10^7C^ ± 0	6.45 × 10^7E^ ± 707
	3	0^L^ ± 0	0^L^ ± 0	0^L^ ± 0	0^L^ ± 0
	5	0^L^ ± 0	0^L^ ± 0	6.51 × 10^7E^ ± 212	4 × 10^7I^ ± 0
M.M; oleoresin	1	5 × 10^7G^ ± 0	3 × 10^7 J^ ± 0	7.05 × 0^7C^ ± 707	7 × 10^7C^ ± 0
	3	0^L^ ± 0	0^L^ ± 0	0^L^ ± 0	0^L^ ± 0
	5	0^L^ ± 0	0^L^ ± 0	0^L^ ± 0	0^L^ ± 0
T.T; essential oil	1	6.65 × 10^7D^ ± 212	4.90 × 10^7H^ ± 134	9.05 × 10^7A^ ± 707	7.01 × 10^7C^ ± 141
	3	2.15 × 10^5L^ ± 212	1.53 × 10^5L^ ± 354	2.5 × 10^5L^ ± 0	2.03 × 10^5L^ ± 353
	5	7.05 × 10^5L^ ± 707	7.05 × 10^5L^ ± 707	6.10 × 10^5L^ ± 141	6.10 × 10^5L^ ± 141
T.T; oleoresin	1	4 × 10^7I^ ± 0	2 × 10^7K^ ± 0	6 × 10^7F^ ± 0	8 × 10^7B^ ± 0
	3	0^L^ ± 0	0^L^ ± 0	0^L^ ± 0	0^L^ ± 0
	5	0^L^ ± 0	1.5 × 0^4L^ ± 7071	0^L^ ± 0	0^L^ ± 0

Means with different superscript are significantly different.

M.M = *Monodora myristica,* T.T = *Tetrapleura tetraptera.*

**Table 5:** DPPH radical scavenging activity of essential oils and oleoresins (%) at different concentrations in *M. myristica* and *T. tetraptera* spices.

Extracts	Concentrations (µg/mL)						
	10	20	40	80	160	320	640
Ascorbic acid	92.60^ab^ ± 1.78	93.20^ab^ ± 2.49	95.82^a^ ± 2.33	93.15^ab^ ± 1.63	92.43^ab^ ± 3.68	90.43^ab^ ± 1.07	87.75^bc^ ± 3.67
M.M essential oil	62.49^klmnop^ ± 17.54	54.01^q^ ± 0.98	53.76^q^ ± 1.87	60.61^mnop^ ± 3.45	64.70^jklmno^ ± 1.50	72.82^fgh^ ± 0.94	79.35^de^ ± 0.17
M.M oleoresin	66.47^ijklm^ ± 1.63	65.41^ijklmn^ ± 2.73	64.85^jklmno^ ± 2.21	66.01^ijklmn^ ± 2.54	70.80^ghi^ ± 1.61	76.38^efg^ ± 1.80	77.09^ef^ ± 2.97
T.T essential oil	59.37^opq^ ± 3.62	58.59^pq^ ± 1.83	61.34^lmnop^ ± 1.40	60.18^nop^ ± 1.39	61.05^lmnop^ ± 1.43	57.25^pq^ ± 2.75	56.75^pq^ ± 1.38
T.T oleoresin	68.38^hijk^ ± 3.21	66.77^ijkl^ ± 2.76	70.54^ghij^ ± 0.91	74.52^efg^ ± 1.09	83.89^ cd^ ± 0.91	92.35^ab^ ± 1.46	93.46^ab^ ± 0.92

Means with the same superscript are not significantly different.

M.M = *Monodora myristica,* T.T = *Tetrapleura tetraptera.*

### Antioxidant capacity of essential oil and oleoresin

The DPPH radical scavenging activity of essential oils and oleoresins (%) at different concentrations in *M. myristica* and *T. tetraptera* spices can be seen in Table 5. In general, the extracts showed promising scavenging activity with a maximum of 93.46% and a minimum of 53.76%. DPPH radical scavenging activity increased with concentration except for *T. tetraptera* essential oil. The oleoresin of *T. tetraptera* showed the better DPPH radical scavenging activity across samples’ concentrations. For example, the highest concentration (640 µg/mL) of *T. tetraptera* oleoresin obtained maximum scavenging power (93.46%) that appeared significantly higher (p < 0.05) compared to the others, which specifically demonstrates enhanced antioxidant properties. Previous reports show roughly 29–37.7% scavenging powers of *M. myristica* seeds could concur with maximum concentration of 120 µg/mL^35^, contrasting 10–41% when concentration is maximum at 400 µg/mL^7^. Lower DPPH scavenging has been recorded at *T. tetraptera* fruit and bark ethanolic and hydroethanolic extracts^1,4,36^. Typically through the reduction of DPPH, the radical scavenging compounds (OH, O_2_^−^, L., LOO., LO.,) act like an antioxidant during lipid oxidation, given the ability to donate an electron or hydrogen molecule^37^. Of all reactive oxygen species, the OH radical appears the stronger, and able to react with most biological molecules found in living cells. Among effective defences of living body against various diseases is the removal of hydroxyl radicals^38^.

The reducing power activity (%) of essential oils and oleoresins at different concentrations in *M. myristica* and *T. tetraptera* spices can be seen in Table 6. Like the DPPH radical scavenging, the reducing power activity would increase with concentration, and more evident at the essential oil of *M. myristica* of this study. Specifically, *M. myristica* essential oil obtained the peak highest reducing power with mean absorbance value of 54.69 at concentration of 640 µg/mL. Despite the higher values at concentrations higher than 100 µg/mL^7^, the ethanol extract of *T. tetraptera* fruit peel (at similar concentrations higher than 100 µg/mL) might possess the lesser reducing power^4^, although the higher reducing power for ethanolic and hydroethanolic extracts of fruit and bark could resemble^1^. Acting as a defense mechanism against lipid peroxidation, the antioxidants in plant materials would bind to metal ions by reducing transition metals such as Fe^2+^ or Cu^+^^33^. Further, the reducing power of essential oils and oleoresins take place largely because the reductones can transfer electrons or hydrogen atoms that break the free radical chain^4^. The total antioxidant capacity (TAC) of essential oils and oleoresins (%) at different concentrations in *M. myristica* and *T. tetraptera* spices can be seen in Table 7. Like the DPPH radical scavenging and reducing power activity, the TAC would also increase with concentration, being more evident at the oleoresin of *M. myristica* of this study. The TAC of both essential oils and oleoresins can be said to be dose dependent. *M. myristica* essential oil obtained the peak TAC value (15.75) at 640 µg/mL concentration, significantly higher (p < 0.05) than that of 360 µg/mL (TAC value = 8.76). *T. tetraptera* oleoresin obtained the second-highest absorbance values, while *M. myristica* oleoresins had the least absorbance levels*.* The TAC recorded in this current work seemed higher than those of *M. myristica*^35^, including those of aqueous and ethanolic extracts of fruits, pulps, and seeds of *T. tetraptera*^8^. Nonetheless and for emphasis, the TAC assay is based on the ability of a reducing agent (antioxidant) to reduce molybdenum (VI) ions to molybdenum (V) giving a green phosphomolybdate (V) complex at 765 nm^20,37,39^.

**Table 6:** Reducing power activity of essential oils and oleoresins (%) at different concentrations in *M. myristica* and *T. tetraptera* spices.

Extracts	Concentrations (µg/mL)						
	10	20	40	80	160	320	640
Gallic acid	0.05^g^ ± 0.02	0.25^rfg^ ± 0.32	0.13^fg^ ± 0.00	0.26^efg^ ± 0.01	0.54^efg^ ± 0.01	1.00^defg^ ± 0.02	2.18^de^ ± 0.05
M.M essential oil	0.04^g^ ± 0.00	0.08^fg^ ± 0.00	0.19^d^ ± 0.01	0.75^efg^ ± 0.16	2.86^efg^ ± 0.69	13.11^b^ ± 2.50	54.69^a^ ± 6.80
M.M oleoresin	0.03^g^ ± 0.00	0.07^fg^ ± 0.03	0.11^fg^ ± 0.00	0.16^fg^ ± 0.06	0.52^efg^ ± 0.02	2.09^def^ ± 0.07	8.04^c^ ± 0.23
T. T essential oil	0.03^ g^ ± 0.00	0.07^ fg^ ± 0.00	0.15^ fg^ ± 0.00	0.26^efg^ ± 0.02	0.55^efg^ ± 0.02	1.05^defg^ ± 0.06	1.91^defg^ ± 0.02
T. T oleoresin	0.04^ g^ ± 0.00	0.07^ fg^ ± 0.00	0.09^ fg^ ± 0.00	0.17^efg^ ± 0.00	0.43^efg^ ± 0.04	1.40^defg^ ± 0.21	7.95^c^ ± 0.45

Means with different superscript are significantly different.

M.M = *Monodora myristica*, T.T = *Tetrapleura tetraptera.*

**Table 7:** Total antioxidant capacity of essential oils and oleoresins (%) at different concentrations in *M. myristica* and *T. tetraptera* spices.

Samples	Concentrations (µg/mL)						
	10	20	40	80	160	320	640
Ascorbic acid	0.04^ h^ ± 0.01	0.04^ h^ ± 0.01	0.03 ± 0.00	0.05^ h^ ± 0.00	0.06^ h^ ± 0.00	0.06^ h^ ± 0.00	0.07^ h^ ± 0.01
M.M oil	0.30^ h^ ± 0.39	0.15^ h^ ± 0.01	0.32^ h^ ± 0.02	0.72^ h^ ± 0.04	1.40^fgh^ ± 0.10	3.23^ef^ ± 0.07	5.81^ cd^ ± 2.45
M.M oleoresin	0.11^ h^ ± 0.02	0.29^ h^ ± 0.01	0.80^gh^ ± 0.05	2.05^fgh^ ± 0.34	4.37^de^ ± 1.63	8.76^b^ ± 3.14	15.75^a^ ± 5.51
T. T oil	0.07^ h^ ± 0.00	0.14^ h^ ± 0.01	0.29^ h^ ± 0.05	0.44^ h^ ± 0.21	1.12^fgh^ ± 0.09	2.87^efg^ ± 0.12	6.10^ cd^ ± 0.56
T.T oleoresin	0.08^ h^ ± 0.00	0.14^ h^ ± 0.02	0.32^ h^ ± 0.02	0.67^ h^ ± 0.06	1.42^fgh^ ± 0.01	2.93^ef^ ± 0.29	6.97^bc^ ± 2.96

Means with different superscript are significantly different.

M.M = *Monodora myristica*, T.T = *Tetrapleura tetraptera.*

## Conclusions

In this study and from *M. myristica* and *T. tetraptera* spices found in the Southeast of Nigeria, both essential oil and oleoresin were secured, respectively, by steam distillation and maceration. Very useful compounds were obtained, through which some considerable antibacterial and antioxidant potentials were exhibited. Essential oil of *M. myristica* appeared to have enhanced antibacterial and antioxidant activity compared to the others. The *T. tetraptera*, however, seemed less effective to inhibit both *E. coli* and *S. aureus* populations. The extraction process, environment, and genetics would be crucial influences on the natural compounds found in each spice’s essential oil and oleoresin, which would in turn strongly impact (both *M. myristica* and *T. tetraptera* spices’) antibacterial and antioxidant capabilities, and hold great promise in food preservation, especially against the autoxidation and microbial aspects of (food) spoilage. Considering the findings of this current work, the direction of future investigations should be to establish the purity of these chemical constituents of essential oil and oleoresin obtained from *M. myristica* and *T. tetraptera* spices, and their corresponding cytotoxic activities, which could possibly help better the understanding that underpin their pharmacological and other associated/related effects.

## Data Availability

The datasets generated during and/or analyzed during the current study are available from the corresponding author upon reasonable request.
